# Effects of Interrupting Prolonged Sitting with Light-Intensity Physical Activity on Inflammatory and Cardiometabolic Risk Markers in Young Adults with Overweight and Obesity: Secondary Outcome Analyses of the SED-ACT Randomized Controlled Crossover Trial

**DOI:** 10.3390/biom14081029

**Published:** 2024-08-19

**Authors:** Sascha W. Hoffmann, Janis Schierbauer, Paul Zimmermann, Thomas Voit, Auguste Grothoff, Nadine B. Wachsmuth, Andreas Rössler, Tobias Niedrist, Helmut K. Lackner, Othmar Moser

**Affiliations:** 1 Division of Theory and Practice of Sports and Fields of Physical Activity, BaySpo—Bayreuth Center of Sport Science, University of Bayreuth, 95440 Bayreuth, Germany; 2 Division of Exercise Physiology and Metabolism, BaySpo—Bayreuth Center of Sport Science, University of Bayreuth, 95440 Bayreuth, Germany; janis.schierbauer@uni-bayreuth.de (J.S.); paul.zimmermann@uni-bayreuth.de (P.Z.); thomas.voit@uni-bayreuth.de (T.V.); auguste.grothoff@uni-bayreuth.de (A.G.); nadine.wachsmuth@uni-bayreuth.de (N.B.W.); 3 Department of Physiology and Pathophysiology, Medical University of Graz, 8010 Graz, Austria; andreas.roessler@medunigraz.at (A.R.); helmut.lackner@medunigraz.at (H.K.L.); 4 Clinical Institute of Medical and Chemical Laboratory Diagnostics, Medical University of Graz, 8010 Graz, Austria; tobias.niedrist@medunigraz.at; 5 Interdisciplinary Metabolic Medicine Trials Unit, Department of Internal Medicine, Division of Endocrinology and Diabetology, Medical University of Graz, 8010 Graz, Austria

**Keywords:** low-grade inflammation, cardiometabolic risk, sedentary behavior, light-intensity physical activity snacks, overweight, obesity, young adults

## Abstract

Sedentary behavior (SB) is an essential risk factor for obesity, cardiovascular disease, and type 2 diabetes. Though certain levels of physical activity (PA) may attenuate the detrimental effects of SB, the inflammatory and cardiometabolic responses involved are still not fully understood. The focus of this secondary outcome analysis was to describe how light-intensity PA snacks (LIPASs, alternate sitting and standing, walking or standing continuously) compared with uninterrupted prolonged sitting affect inflammatory and cardiometabolic risk markers. Seventeen young adults with overweight and obesity participated in this study (eight females, 23.4 ± 3.3 years, body mass index (BMI) 29.7 ± 3.8 kg/m^2^, glycated hemoglobin A1C (HbA_1c_) 5.4 ± 0.3%, body fat 31.8 ± 8.2%). Participants were randomly assigned to the following conditions which were tested during an 8 h simulated workday: uninterrupted prolonged sitting (SIT), alternate sitting and standing (SIT-STAND, 2.5 h total standing time), continuous standing (STAND), and continuous walking (1.6 km/h; WALK). Each condition also included a standardized non-relativized breakfast and lunch. Venous blood samples were obtained in a fasted state at baseline (T_0_), 1 h after lunch (T_1_) and 8 h after baseline (T_2_). Inflammatory and cardiometabolic risk markers included interleukin-6 (IL-6), c-reactive protein (CRP), total cholesterol (TC), high-density lipoprotein cholesterol (HDL-C), low-density lipoprotein cholesterol (LDL-C), triglycerides (TGs), visceral fat area (VFA), triglyceride-glucose (TyG) index, two lipid ratio measures, TG/HDL-C and TC/HDL-C, albumin, amylase (pancreatic), total protein, uric acid, and urea. We found significant changes in a broad range of certain inflammatory and cardiometabolic risk markers during the intervention phase for IL-6 (*p* = 0.014), TG (*p* = 0.012), TC (*p* = 0.017), HDL-C (*p* = 0.020), LDL-C (*p* = 0.021), albumin (*p* = 0.003), total protein (*p* = 0.021), and uric acid (*p* = 0.040) in favor of light-intensity walking compared with uninterrupted prolonged sitting, alternate sitting and standing, and continuous standing. We found no significant changes in CRP (*p* = 0.529), creatinine (*p* = 0.199), TyG (*p* = 0.331), and the lipid ratios TG/HDL-C (*p* = 0.793) and TC/HDL-C (*p* = 0.221) in response to the PA snack. During a simulated 8 h work environment replacement and interruption of prolonged sitting with light-intensity walking, significant positive effects on certain inflammatory and cardiometabolic risk markers were found in young adults with overweight and obesity.

## 1. Introduction

Obesity is associated with adverse health outcomes and increased cardiovascular morbidity and mortality throughout the life course [1,2,3]. Over the years, the prevalence of overweight and obesity has dramatically increased across all age groups and countries [4]. Global estimates for overweight and obesity suggest that over 4 billion people may be affected by the year 2035, compared with over 2.6 billion in 2020, representing a theoretical increase from 38% to over 50% within the next decade [5]. 

An energy surplus, physical inactivity, and sedentary behavior (SB) are dominant factors in the obesogenic environment and lead to an accumulation of visceral fat (VF) in the adipose tissue [6]. VF is primarily associated with the development of type 2 diabetes (T2D), cardiovascular disease (CVD), and different types of cancers [7,8,9]. Furthermore, it is a major driver for developing a low-grade local inflammation in adipose tissue, which in turn contributes to the pathogenesis of multiple diseases, which are summarized as metabolic syndrome [10]. Therefore, for the early identification and progression of different cardiovascular diseases, certain inflammatory biomarkers are increasingly recognized to have relevant clinical value [11]. 

Systemic inflammation involves M1 macrophages in the adipose tissue releasing inflammatory markers like interleukin-6 (IL-6), tumor necrosis factor alpha (TNF alpha), and C-reactive protein (CRP) [3,10]. As a result, low-grade inflammation has been linked to the development of insulin resistance, neurodegeneration, and atherosclerosis [7,8,12]. Moreover, physical inactivity and SB further fuel the risk of these conditions [13,14,15,16]. Further research suggested that SB, defined as any waking behavior characterized by an energy expenditure ≤1.5 metabolic equivalents of task (METs), while in a sitting, lying, or reclining posture [17], is an independent risk factor of several health outcomes [18,19,20]. Furthermore, high and/or prolonged SB volumes were found to be more prevalent in young adults [21,22,23,24]. 

In contrast, previous studies reported that regular physical activity (PA) has chronic anti-inflammatory effects [3,25,26]. Furthermore, PA can reduce abdominal and visceral fat, and regular PA influences the release of anti-inflammatory cytokines [3,26,27]. IL-6, as a key myokine, appears to play a pivotal role in this context [26]. IL-6 initially has an acute pro-inflammatory effect, and it can increase up to 100-fold on both a cellular and systemic level during a sufficient PA load and decreases back to baseline within 1 h after exercise [3,25,28]. The PA-induced increase in IL-6 levels is therefore responsible for a subsequent rise in circulating levels of anti-inflammatory cytokines such as IL-10 and IL-1 receptor antagonist (IL-1ra) and further suppresses the production of TNF alpha and IL-1 beta [3,25,26,28]. TNF is, in turn, responsible for insulin resistance in humans [29], and exercise may protect against TNF-induced insulin resistance in part due to its anti-inflammatory effects mediated by muscle-derived IL-6 [3,25]. 

Besides this inflammatory cascade, however, threatening circumstances for the development of CVD, like plaque formation and the emergence of fatty streaks, already start at a young age, and their progression is influenced by several lipid metabolism parameters like elevated triglycerides (TGs), total cholesterol (TC), low-density lipoprotein cholesterol (LDL-C), and the ratio of triglyceride vs. high-density lipoprotein cholesterol (TG/HDL-C ratio) [30,31]. In addition, obesity increases the risk of dyslipidemia, and atherogenic dyslipidemia in turn belongs to the conditions of metabolic syndrome [32,33]. 

In addition, early stages of chronic kidney disease and high levels of uric acid are associated with an increased risk of CVD, whereas, furthermore, abnormal glucose, lipid metabolism, oxidative stress, and inflammatory responses are linked to diabetic kidney disease, which is one of the most common complications of diabetes [34,35]. 

Although certain well-known inflammatory and cardiometabolic markers play a key role in exercise metabolism, specific mechanisms linking SB and different types of light-intensity PA to markers of inflammation and cardiometabolic risk remain unclear. 

Therefore, within this secondary outcome analysis, we assess the effects of interrupting prolonged SB with different types of light-intensity PA snacks (LIPASs; alternate sitting and standing, walking or standing continuously) on inflammatory and cardiometabolic risk markers during an 8 h simulated workday in young adults with overweight and obesity. 

## 2. Materials and Methods

The SED-ACT study was a single-center randomized controlled four-arm crossover trial that was undertaken between February and July 2023 at the University of Bayreuth (GER) assessing the effects of how a change in body position with light-intensity PA snacks (alternate sitting and standing, walking or standing continuously) compared to uninterrupted prolonged sitting affects inflammatory and physiological processes. A detailed description of the study and the results of the primary outcome have been published recently [24]. This trial was planned and carried out in accordance with the principles of Good Clinical Practice and the Declaration of Helsinki [36]. The local ethics committee of the University of Bayreuth (Germany) approved the study protocol (O 1305/1-GB, 13 December 2022), and this trial was registered with the German Clinical Trial Register (DRKS00031425). Before any trial-related activities were performed, participants had to provide written informed consent.

### 2.1. Eligibility Criteria 

Recruiting took place via notices (digital and paper-based), via the homepages of several organizational units of the University of Bayreuth, and if necessary, through social media. The following main eligibility criteria were defined and assessed by an investigator during a joint information and preparation meeting one week prior to the screening visit: age between 18 and 29 years (both age groups included) and classified as overweight or obese according to the WHO criteria with a BMI ≥ 25.0 kg/m^2^. Exclusion criteria included simultaneous enrollment in a different study, acute infection due to COVID-19, serious acute/chronic illnesses that preclude participation in the study, (orthopedical) restrictions that prevent sitting, standing, or walking for more than 8 h, and if a BMI greater than 25.0 kg/m^2^ was obviously classified due to increased muscle mass and no apparent overweight. In addition, participants who were taking medication on a long-term and acute basis, which would influence the study results, were excluded from the study. Females who were pregnant were also excluded. 

### 2.2. Study Design 

**Figure 1:**
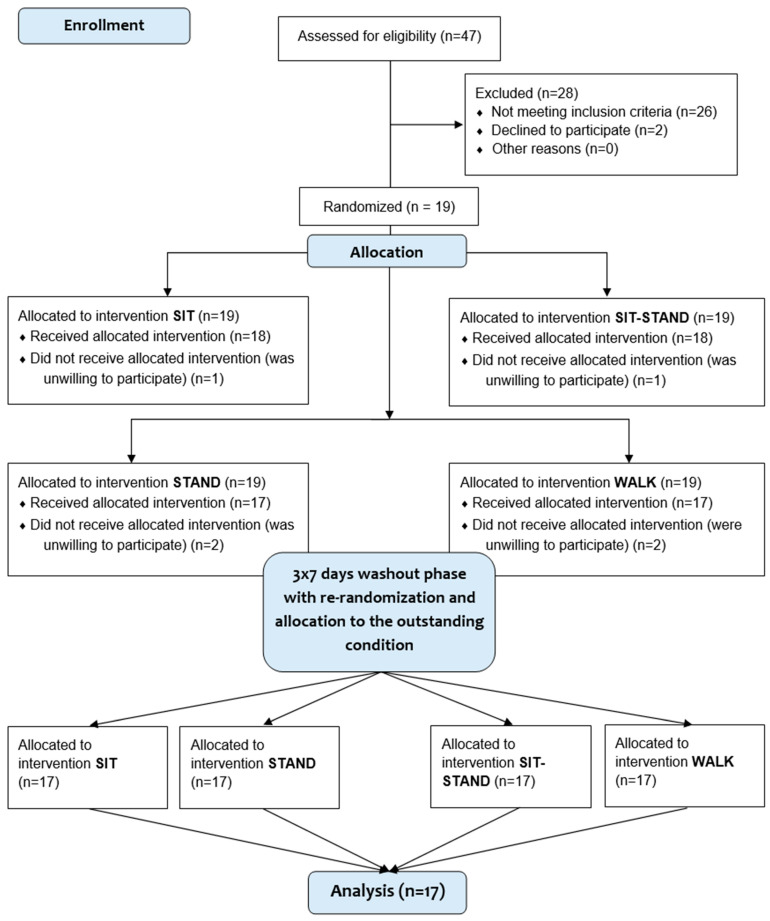
Participant flow chart.

After enrolment in the study, participants were randomized to the order of simulated work conditions by a research associate that was not other involved in the study (performed by Research Randomizer^®^ 4.0 [Social Psychology Network, Lancaster, PA, USA], 1:1:1:1) [37]. Participants took part in an initial screening visit and completed four 8 h simulated work and learning conditions in random order: (1) uninterrupted prolonged sitting (SIT), (2) alternate sitting and standing (SIT/STAND), (3) continuous standing (STAND), and (4) continuous walking (WALK). Always with one week in between, participants were tested within the remaining trial arms. The initial screening visit and each trial intervention were conducted at our research lab at the Bayreuth Center of Sport Science (BaySpo) of the University of Bayreuth. As shown in the CONSORT flow diagram [38] (Figure 1), out of 47 people screened, 19 consented and were randomized, and 28 participants were withdrawn for not meeting the eligibility criteria. Among those randomized, 2 withdrew their participation during the first intervention for personal reasons. The final sample consisted of 17 young adults with overweight and obesity.

### 2.3. Screening Visit

Prior to the start of the screening visit and each trial visit, participants had to fast for at least 12 h and refrain from any strenuous PA for at least 24 h. They were also not allowed to consume alcohol within 24 h before each visit. Participants’ body composition was evaluated via bioelectrical impedance analysis (BIA; Inbody 720, Inbody Co., Seoul, Republic of Korea), and their body heights were measured manually (Seca 217, Seca, Hamburg, Germany). During BIA, the following domains were analyzed: body weight, intra- (ICW) and extracellular water (ECW), total body water (TBW), skeletal muscle mass (SMM), body fat mass (FM), and visceral fat area (VFA). To clarify any abnormalities in the blood and to verify whether their participants’ glucose metabolism was impaired, complete blood count and glycated hemoglobin A1C (HbA1_c_) levels were assessed with a venous blood sample from the antecubital vein. HbA_1c_ values below 39 mmol/mol (5.7%) were considered normal, while values of 39 to <48 mmol/mol (5.7 to <6.5%) were considered borderline, and HbA_1c_ values of ≥48 mmol/mol (≥6.5%) led to the classification of type 2 diabetes [39]. Finally, an overnight fasted oral glucose tolerance test (OGTT) was conducted at 7:30 a.m. during which participants consumed a 75 g glucose solution (Glucoral^®^ 75 citron, Germania Pharmazeutika, Vienna) in 300 mL of water according to current guidelines. Participants were required to remain seated throughout the test. Then, 20 μL capillary blood samples were drawn from a hyperemized earlobe before and 30, 60, 90, and 120 min after consumption to analyze glucose metabolism (Biosen S-Line Lab+, EKF diagnostics, Barleben, Germany).

### 2.4. Trial Visits

Before each trial visit, participants had to fast for at least 12 h and refrain from any strenuous PA and were also not allowed to consume alcohol for at least 24 h. Further, participants were advised that each trial visit would require them to eat the same meal the evening before. We discussed appropriate evening meals with the participants to accomplish this, and participants were then advised to consume approximately 1 g of carbohydrates per kilogram of body weight. This had to be replicated accordingly during the following trial visits. Each trial visit began with participants being asked whether they consumed the same meal and amount as the previous ones. For all trial days, participants received a standardized non-relativized breakfast. For breakfast, all the participants received the same standardized meal; however, for lunch they were allowed to choose between two different meals. Each person consumed the same meal each trial visit. Breakfast and lunch were consumed between 08.30 and 09.00 a.m. and between 12.00 and 12.30 p.m., respectively. Both meals were brought and served directly to the participants so that they were able to remain in the corresponding type of activity. Participants were asked to consume each meal within 15 min. A description of the macronutrients of the served meals has been previously published [24]. Participants were allowed to drink water and sugar-free drinks during the intervention phase. During the 8 h intervention period, participants were allowed to read, watch movies, work, or study on the computer in the respective setting.

**Figure 2:**
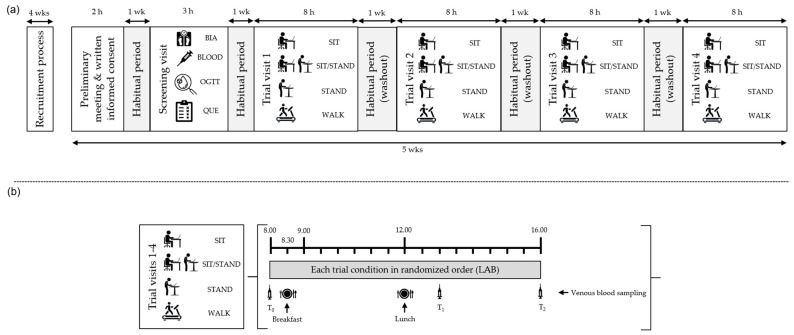
Study protocol: (**a**) overview of the study design; (**b**) overview of the trial visits. Participants (*n* = 17) completed four trial visits in a randomized order separated by one week. Venous blood samples were collected fasted at T_0_, T_1_, and T_2_. Meals were provided at 08.30 a.m. and 12.00 p.m. BIA, bioelectric impedance analysis; OGTT, oral glucose tolerance test; QUE, questionnaire; SIT, uninterrupted prolonged sitting; SIT/STAND, alternate sitting and standing; STAND, continuous standing; WALK, continuous walking.

During SIT, participants remained seated for an 8 h period but were allowed to use the toilet at the following times: before 08.30 a.m., between 10.00 and 10.30 a.m., during lunch time (12.00–12.30 p.m.) and between 15.00 and 16.00 p.m., but no other PA was permitted. During STAND, participants were required to stand continuously using a height-adjustable office desk (Aeris^®^ Active Office, Aeris GmbH, Haar, Germany). During SIT/STAND, participants were asked to change from sitting to standing at the same Aeris^®^ Active Office height-adjustable work desk each hour at progressively longer intervals throughout the day: for 10 min at 09.20 a.m. and 10.20 a.m., for 15 min at 11.30 a.m. and during lunch time (12.00–12.30 p.m.), for 20 min at 13.40 p.m. and 14.20 p.m., and for 30 min at 15.00 and 16.00 p.m. This resulted in 2.5 h of standing per day and followed previous studies that investigated the effects of alternating between sitting and standing and light-intensity walking on ambulatory blood pressure, glucose levels, and musculoskeletal discomfort [40,41,42]. During WALK, participants were required to work at a normal treadmill with a special shelf for books, tablets, and computers (LifeFitness Platinum Series, Life Fitness Europe, Unterschleißheim, Germany) in a slow walking activity (1.6 km/h; Figure 2). Exceptions for visiting the toilet during SIT-STAND, STAND, and WALK were made at the same times as during the SIT condition.

### 2.5. Blood Sampling

A standard gauge cannula was placed into a subcutaneous vein for blood sampling. To prevent blood clotting in the cannula, it was occasionally flushed with sterile 0.9% saline solution. Venous blood samples at each trial visit were obtained fasted at baseline (T_0_), 1 h after lunch (T_1_) [43], and 8 h (T_2_) and dispensed evenly into lithium heparin tubes (BD Vacutainer^®^ SST^TM^ II Advance; BD Belliver Industrial Estate, Plymouth, UK). The blood serum vacutainer was left to rest for a minimum of 30 min prior to being centrifuged at room temperature for 10 min at 3500× *g*. The serum was then aliquoted and stored at −80 °C at the research facility. After the last trial visit was finished, serum samples were analyzed in a single batch. All analyses were performed on a cobas 8000 analyzer (Roche Diagnostics GmbH, Mannheim, Germany) with standardized assays by the same manufacturer, calibrated to international standards.

### 2.6. Inflammatory and Cardiometabolic Risk Markers

For clinical and laboratory evaluation data of inflammatory and cardiometabolic risk markers including interleukin-6 (IL-6), C-reactive protein (CRP), total cholesterol (TC), high-density lipoprotein cholesterol (HDL-C), low-density lipoprotein cholesterol (LDL-C, calculated via the Friedewald equation), triglycerides (TGs), two lipid ratio measures, TG/HDL-C and TC/HDL-C, the triglyceride-glucose (TyG) index, albumin, pancreatic amylase, total protein, uric acid, urea, and creatinine were assessed. Lipid ratios such as triglycerides to HDL cholesterol >3.5 and total cholesterol to HDL cholesterol >5 were considered as predictors of CVD risk [31]. In addition, the TyG index as the product of TG (mg/dL) and fasting glucose (mg/dL) was calculated. Likewise, TyG has shown high accuracy for insulin resistance. The TyG index indirectly assesses IR through a mathematical model that uses only laboratory data on fasting triglyceride and glucose concentrations [44,45].

### 2.7. Statistics

All data were assessed for distribution by means of the Shapiro–Wilk normality test. Data are presented according to their distribution as arithmetic mean (95% CI) or median (interquartile range [IQR]). Pearson or Spearman’s rank correlation was used to study the interplay between body composition and risk markers. The changes in the variables studied in each condition between baseline (T_0_), during condition (T_1_), and after condition (T_2_) were calculated as Δ1 (T_1_−T_0_), Δ2 (T_2_−T_0_), and Δ3 (T_2_−T_1_). Changes were analyzed using one-way analysis of variance (ANOVA) with post hoc Tukey’s multiple comparisons test, the Friedman test, or a mixed-effects model with a post hoc Dunn’s multiple comparisons test between conditions. Data were analyzed in GraphPad Prism Software version 8.0.2 (GraphPad, San Jose, CA, USA). An a priori power analysis was performed for the study (G-Power, v.3.1.9.7, HHU-Düsseldorf, Düsseldorf, Germany) as previously described [24]. Statistical significance was accepted at *p* < 0.05 (two-tailed).

## 3. Results

A total of 17 young adults with overweight and obesity (eight females) completed the screening examination and all four trial arms. The anthropometric characteristics and inflammatory and cardiometabolic risk markers of the study participants in a fasted state during the screening examination are displayed in Table 1.

### 3.1. Results of the Screening Visit

The baseline body composition (BC) parameters as well as the inflammatory and cardiometabolic risk markers during the screening examination prior to the trial visits are presented in Table 1. Regarding anthropometrical characteristics, 58.8% (*n* = 10) of the participants were overweight, and 41.2% (*n* = 7) were obese. Regarding the BC parameters, participants showed a high fat mass (31.8%, 95% CI: 27.6–36.0) as well as a high visceral fat area (119.2 cm^2^, 95% CI: 101.7–136.7) [46,47]. About 59% of the participants showed an unhealthy visceral fat area above 100 cm^2^. The mean LDL-C of 103.0 (95% CI: 90.9–114.8), as a cause of atherosclerotic cardiovascular disease (ASCVD), was slightly increased [48,49].

Table 2 shows bivariate correlations between clinical-laboratory risk markers and BC parameters. Positive correlations were found for IL-6 (*p* = 0.016), TG (*p* = 0.015), TG/HDL-C ratio, and total protein (*p* = 0.0.35) with VFA. BMI (*p* = 0.039) was also positively correlated with IL-6 and with total protein (*p* = 0.015). Uric acid (*p* = 0.041) was positively correlated with age, whereas creatinine (*p* = 0.035) showed an inverse correlation with FM%.

### 3.2. Results of the Trial Visits

**Figure 3:**
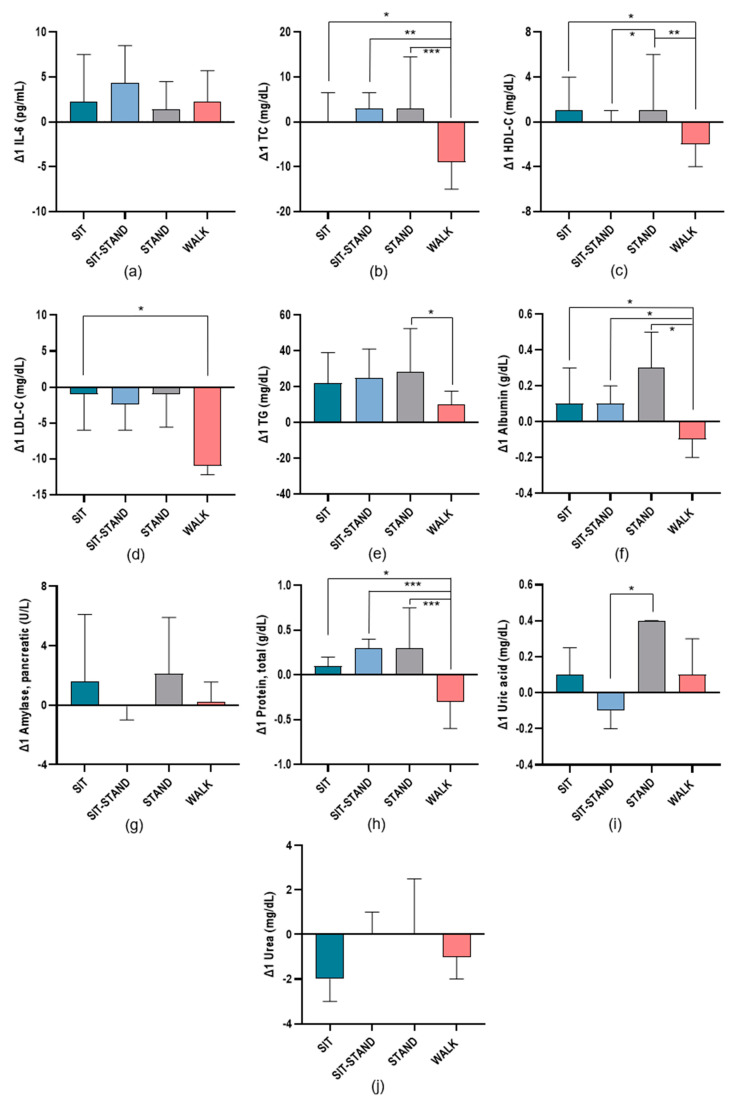
Changes in inflammatory and cardiometabolic risk markers studied in each condition Δ1(T_1_−T_0_) as follows: (**a**) interleukin-6 (IL-6); (**b**) total cholesterol (TC); (**c**) high-density lipoprotein cholesterol (HDL-C); (**d**) low-density lipoprotein cholesterol (LDL-C); (**e**) triglyceride (TG); (**f**) albumin; (**g**) amylase, pancreatic; (**h**) total protein; (**i**) uric acid; (**j**) urea. * *p* ≤ 0.05; ** *p* ≤ 0.01; *** *p* ≤ 0.001.

Figure 3 shows significant changes in inflammatory and cardiometabolic risk markers between baseline and T_1_ in each condition during the trial visits. TC values were significantly lower during WALK compared with SIT (*p* = 0.021), SIT-STAND (*p* = 0.002), and STAND (*p* < 0.001). This can also be seen in the HDL-C values between WALK and STAND (*p* = 0.003) and SIT (*p* = 0.020). There was also a significant difference between STAND and SIT-STAND (*p* = 0.046). Significantly lower LDL-C values were also observed in WALK compared with SIT (*p* = 0.041). Regarding the triglyceride measures, significant differences between STAND and WALK (*p* = 0.012) were observed. Furthermore, there were significant changes in albumin between STAND and WALK (*p* = 0.003), between SIT-STAND and WALK (*p* = 0.033), and between SIT and WALK (*p* = 0.020). Additionally, total protein values were also significantly lower during WALK compared with SIT (*p* = 0.020), SIT-STAND (*p* = 0.001), and STAND (*p* < 0.001). Significantly lower uric acid values were observed in SIT-STAND compared to STAND (*p* = 0.034). Overall, we observed no significant changes at any time in CRP, creatinine, TyG, and the lipid ratios TG/HDL-C and TC/HDL-C in response to the PA snack during the intervention phase.

**Figure 4:**
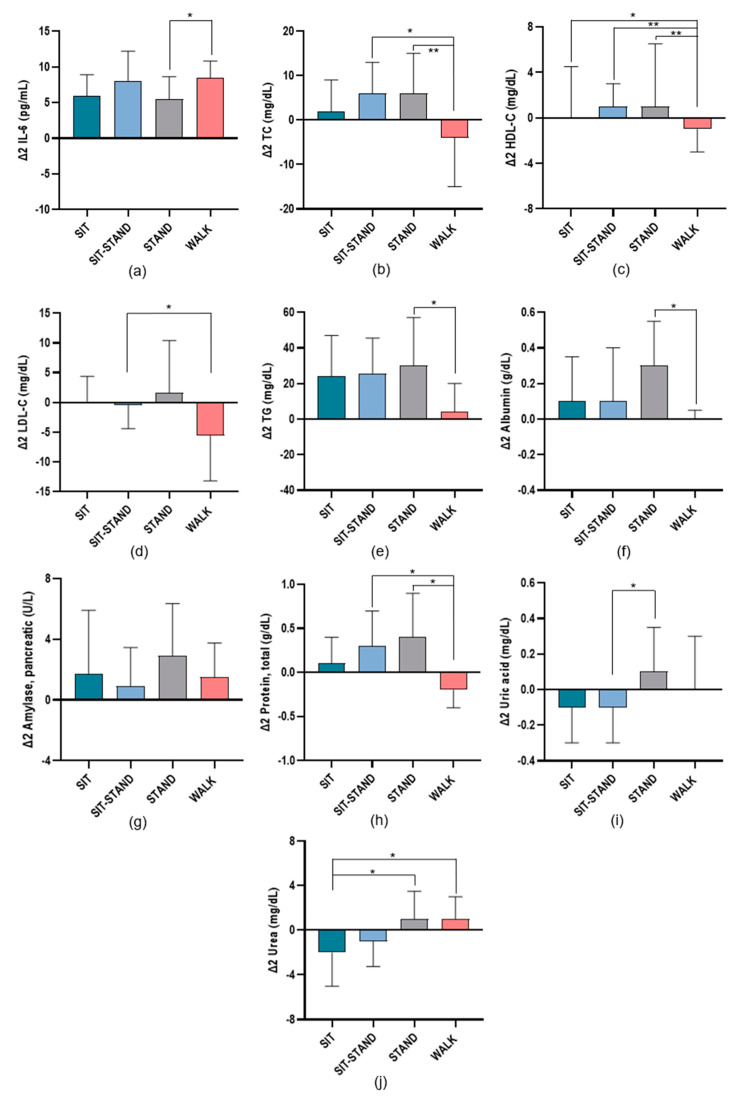
Changes in inflammatory and cardiometabolic risk markers studied in each condition Δ2(T_2_−T_0_) as follows: (**a**) interleukin-6 (IL-6); (**b**) total cholesterol (TC); (**c**) high-density lipoprotein cholesterol (HDL-C); (**d**) low-density lipoprotein cholesterol (LDL-C); (**e**) triglyceride (TG); (**f**) albumin; (**g**) amylase, pancreatic; (**h**) total protein; (**i**) uric acid; (**j**) urea. * *p* ≤ 0.05; ** *p* ≤ 0.01.

Figure 4 shows significant changes in inflammatory and cardiometabolic risk markers between baseline and T_2_ in each condition during the trial visits. For IL-6, a significant effect of WALK was found compared with STAND (*p* = 0.017). Like the observed differences between baseline and T_1_, TC values were significantly lower during WALK compared with SIT-STAND (*p* = 0.012), and STAND (*p* = 0.003), but not compared with SIT.

Also like the above-mentioned differences in the HDL-C values, significant differences between WALK and STAND (*p* = 0.010), SIT-STAND (*p* = 0.010) and SIT (*p* = 0.046) were observed. LDL-C values were also significantly lower in WALK compared with SIT-STAND (*p* = 0.021). TG values were significantly lower during WALK compared with STAND (*p* = 0.040). Albumin values were only significantly lower during WALK compared with STAND (*p* = 0.028). For total protein, significant differences were observed during WALK compared with SIT-STAND (*p* = 0.014) and STAND (*p* < 0.021). Also, significantly lower uric acid values were observed in SIT-STAND compared with STAND (*p* = 0.024). Regarding urea, significant differences between WALK and SIT (*p* = 0.028), and between STAND and SIT (*p* = 0.012) were observed.

**Figure 5:**
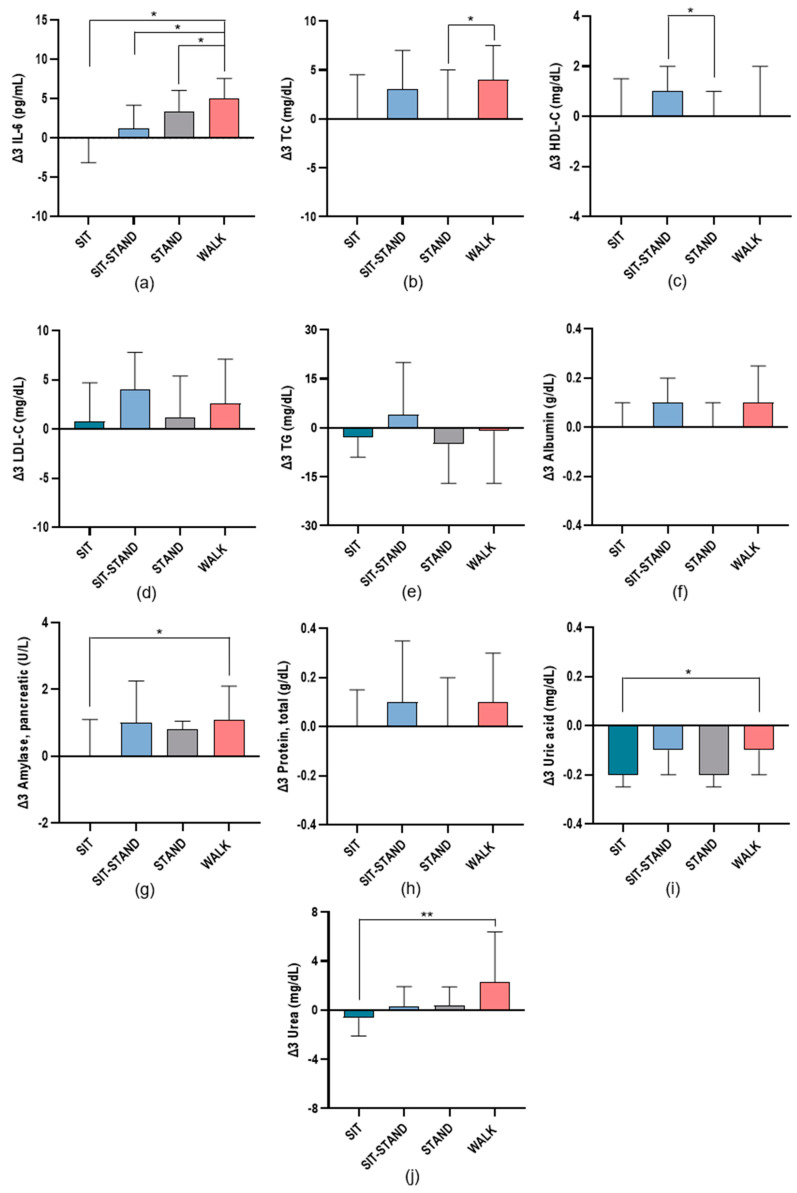
Changes in inflammatory and cardiometabolic risk markers studied in each condition Δ3(T_2_−T_1_) as follows: (**a**) interleukin-6 (IL-6); (**b**) total cholesterol (TC); (**c**) high-density lipoprotein cholesterol (HDL-C); (**d**) low-density lipoprotein cholesterol (LDL-C); (**e**) triglyceride (TG); (**f**) albumin; (**g**) amylase, pancreatic; (**h**) total protein; (**i**) uric acid; (**j**) urea. * *p* ≤ 0.05; ** *p* ≤ 0.01.

Figure 5 shows significant changes in inflammatory and cardiometabolic risk markers between T_2_ and T_1_ in each condition during the trial visits. IL-6 values were significantly higher in WALK compared with STAND (*p* = 0.046), SIT-STAND (*p* = 0.040), and SIT (*p* = 0.014). Regarding TC values, significant differences between WALK and STAND (*p* = 0.017) were observed.

Differences in the HDL-C values were only observed between SIT-STAND and STAND (*p* = 0.024). Amylase values were only significantly different from each other between WALK and SIT (*p* = 0.017). Uric acid values differed significantly between SIT and WALK (*p* = 0.040). Finally, urea values show significant differences between WALK and SIT (*p* = 0.003).

## 4. Discussion

This is the first study that examined the effects of interrupted prolonged sitting by different LIPASs on certain inflammatory and cardiometabolic risk markers during an 8 h simulated workday in young adults with overweight and obesity. The main finding of the present study was that light-intensity walking predominantly had a positive effect on inflammatory and cardiovascular risk markers compared with uninterrupted prolonged sitting, alternate sitting and standing, and continuous standing. While CRP, the lipid ratios TC/HDL-C and TG/HDL-C, the TyG index, and creatinine demonstrated no significant differences between the analyzed trial arms at any time, our results indicated significant changes between T_2_ and T_0_ in IL-6, TC, LDL-C, and TG and between T_2_ and T_1_ in IL-6, pancreatic amylase, uric acid, and urea favoring light-intensity walking instead of standing, alternate sitting and standing, or prolonged sitting.

Our results showed that the level of circulating IL-6 increases in response to the used type and nature of LIPASs. Since the action of muscle-derived IL-6 depends on frequency, duration, and intensity, and the highest increase in IL-6 was found in response to running, here, for the first time, our results showed changes in the elevation of IL-6 during PA which occurs already during light-intensity walking [50]. However, it was stated before that more than 50% of the variation in plasma IL-6 following PA can be explained by the duration of PA alone [25,50]. This also applies to our study results. The longer the walking time, the greater the changes observed in the release of IL-6 during walking compared to STAND, SIT-STAND, and SIT. Furthermore, a significant increase in IL-6 and IL-10 after an acute bout of physical exercise was also observed by others [51]. In contrast, recent research found that IL-6 was not but TNF alpha was affected by a walking exercise; however, the participants were postmenopausal women with obesity (68–72 years old), and this might have affected the study outcomes [52]. Although an increase in circulating IL-6 is an initial pro-inflammatory status, previous studies have demonstrated that the IL-6 response to PA is not preceded by an increase in TNF alpha and further led to an increase in the anti-inflammatory cytokines IL-1ra and IL-10 [25]. The function of IL-1ra is further to inhibit the pro-inflammatory work of IL-1 beta, and IL-10 is responsible for the downregulation of adaptive immune responses and ultimately terminating inflammatory responses [3,53]. In the present study, positive associations were found between VFA and IL-6 (*p* = 0.016). Previous studies have shown similar associations [15,54,55] and demonstrated opposite associations for IL-10 and visceral fat [15]. Additionally, IL-6 exerts its effects peripherally in several organs in a hormone-like fashion to result in improved insulin sensitivity and fatty acid oxidation and increased lipolysis in adipose tissue [25,56].

Comparable to the effects of light-intensity walking on the release of IL-6, our research indicated significant and positive changes during the intervention phase regarding lipid metabolism in normoglycemic young adults with overweight and obesity. TC was significantly lower during WALK compared with all other conditions, and TG was significantly lower during WALK compared with STAND. However, lower levels, but not significant, were also observed compared with SIT and SIT-STAND, respectively, whereas LDL-C was significantly decreased during WALK compared with SIT and SIT-STAND. An earlier study suggested that interrupting prolonged sitting with hourly high-intensity PA breaks improved postprandial triglyceride and HDL-C concentrations [57]. Moreover, further studies on the influence of including regular physical exercise on lipid parameters reported that reducing inactivity by even increasing the time spent walking or standing is more effective than one hour of PA compared with prolonged sitting in improving triglyceride parameters [58]. However, in our study, no positive changes were observed in HDL-cholesterol. Additionally, after including LIPASs or other exercise treatments, improvements in metabolic profile were found in patient groups or older adults; however, most of the interventions carried out only showed initial effects after 12 weeks of treatment [59,60,61]. Our study showed that even acute continuous light-intensity slow walking over an 8 h period significantly improved important lipid parameters already in young adults. This was also confirmed by others [62], who found that light-intensity walking by using a treadmill significantly improved cardiometabolic risk levels compared with prolonged sitting.

Regarding albumin, our results showed significant alterations in serum albumin during WALK compared with all other conditions. Albumin is synthesized by the liver, and it is well known as a muscle-related parameter [63,64] that is affected by several mechanisms and often examined in the context of sarcopenia at older age [64]. Serum albumin is stored extracellularly in muscles and leaks into the muscle cells immediately after exercise. However, the role of serum albumin in connection with muscle performance is not fully understood. It has been reported that low serum albumin could lead to muscle breakdown in older ages [65,66], maybe due to an acute inflammatory process similar to an increase in IL-6 and pancreatic amylase, and on the other hand, albumin could be part of several pathways leading to muscle hypertrophy [67]. In contrast to previous research, which investigated a cohort of healthy young adults, in our study, serum albumin was not associated with measures of relative muscle mass [64].

Increased levels of serum uric acid (SUA) often lead to the occurrence of gout, and hyperuricemia is aggravated with the increase in SUA levels [68,69]. Our observations show SUA levels were highest during STAND compared with the other conditions, whereas significant differences were only observed compared with SIT-STAND and for WALK compared with SIT during the time between T_2_ and T_1_. Our results can be confirmed by previous research, where it was pointed out that regular exercise can significantly reduce SUA, and participants who were sedentary for more than 10 h per day were more likely to develop hyperuricemia than people who were less sedentary (<5 h per day) [68,70]. Additionally, PA is a valuable alternative to pharmacotherapy to reduce the increased risk of mortality due to high SUA [71]. However, there is no uniform standard for PA dose and intensity at present for preventing or treating hyperuricemia [68].

The strength of the present study is that it provides novel evidence in replacing uninterrupted prolonged sitting with LIPASs to positively affect several inflammatory and cardiometabolic risk markers in young adults. A further strength is its randomized crossover design, which allows control of within-participant factors across experimental exposures and thereby improves internal validity and reliability. In contrast, our reporting has diverse limitations. First, the number of included participants is relatively small, though the obtained data of this study should be considered relevant for hypothesis-generating and to verify the evidence base. Secondly, LDL-C was not directly measured. However, calculated LDL-C provides about 6–7 mg/dL lower values compared with direct LDL-C measurements in both genders [30]. Thirdly, only acute effects of inflammatory and cardiometabolic risk factors were investigated, and this study cannot be used to extrapolate the long-term effects of the respective interventions.

## 5. Conclusions

These novel findings from a young and high-risk population for SB suggest that replacing uninterrupted prolonged sitting with light-intensity slow walking positively influences markers associated with inflammation and cardiometabolic importance. In addition to the existing WHO guidelines on physical activity of at least 150 min of moderate-intensity PA per week, we recommend integrating LIPASs (e.g., walking) into every individual daily routine as often as possible, especially within the workplace. All PA counts, since any amount is better than none. Further experimental evidence is needed to confirm these results in other populations and to determine the mechanisms linking SB and PA to inflammation and cardiometabolic health in more detail.

## Figures and Tables

**Table 1:** Baseline anthropometric and clinical-laboratory characteristics of the study participants.

Characteristics	Mean (95% CI, *n* = 17)	Inflammatory and Cardiometabolic Risk Markers	Mean (95% CI, *n* = 17)
Females (*n* [%]) *	8 (47.1)	IL-6 (pg/mL)	3.2 (1.7–4.8)
Age (years)	23.4 (21.7–25.0)	CRP (mg/L	2.6 (0.7–4.5)
Height (cm)	173.8 (167.5–180.0)	TC (mg/dL)	180.0 (166.2–192.7)
Weight (kg)	90.1 (80.7–99.5)	HDL-C (mg/dL)	54.1 (47.9–60.2)
BMI (kg/m^2^)	29.7 (27.8–31.6)	LDL-C (mg/dL	103.0 (90.9–114.8)
*Body Composition*		TG (mg/dL)	112.6 (92.7–132.5)
ICW (L)	28.3 (24.7–31.8)	TC/HDL-C (mg/dL)	3.4 (3.1–3.8)
ECW (L)	16.8 (14.7–18.9)	TG/HDL-C (mg/dL)	2.2 (1.7–2.7)
TBW (L)	45.1 (39.4–50.8)	LDL-C/HDL-C (mg/dL)	2.0 (1.7–2.3)
FM (kg)	29.2 (23.9–34.4)	TyG index (mg/dL)	8.4 (8.2–8.6)
FM (%)	31.8 (27.6–36.0)	Albumin (g/dL)	4.9 (4.7–5.0)
SMM (kg)	34.9 (30.2–39.5)	Amylase, pancreatic (U/L)	24.3 (20.3–28.4)
SMM (%)	38.6 (36.0–41.1)	Protein, total (g/dL)	7.6 (7.4–7.7)
VFA (cm^2^)	119.2 (101.7–136.7)	Uric acid (mg/dL)	5.8 (5.2–6.4)
BMR (kcal)	1700.0 (1531.0–1868.0)	Urea (mg/dL)	24.3 (20.8–27.8)
		Creatinine (mg/dL)	0.9 (0.8–1.0)

ICW, intracellular water; ECW, extracellular water; TBW, total body water; FM, fat mass; SMM, skeletal muscle mass; BMR, basal metabolic rate; VFA, visceral fat area; IL-6, interleukin-6; CRP, C-reactive protein; TC, total cholesterol; HDL-C, high-density lipoprotein cholesterol, LDL-C, low-density lipoprotein cholesterol; TG, triglyceride; TC/HDL-C, total cholesterol/high-density lipoprotein cholesterol ratio; TG/HDL-C, triglyceride/high-density lipoprotein cholesterol ratio; LDL-C/HDL-C, low-density lipoprotein cholesterol/high-density lipoprotein cholesterol ratio; TyG index, triglyceride-glucose index. * Proportion of females is given as an absolute number with a relative value in %. Data are presented as means (95% CI), unless otherwise noted.

**Table 2:** Bivariate correlations between clinical-laboratory risk markers and body composition (BC) parameters of the study participants.

	Age (Years)	BMI (kg/m^2^)	FM (%)	SMM (%)	VFA (cm^2^)
IL-6 (pg/mL)	0.266	0.510	0.297	0.183	0.583
	(0.298)	(0.039 *)	(0.245)	(0.478)	(0.016 *)
CRP (mg/L)	0.475	0.319	0.070	0.057	0.460
	(0.056)	(0.209)	(0.788)	(0.827)	(0.065)
TC (mg/dL)	0.020	−0.144	−0.120	−0.211	−0.213
	(0.941)	(0.661)	(0.645)	(0.415)	(0.410)
HDL-C (mg/dL)	−0.168	−0.087	0.369	0.154	−0.178
	(0.513)	(0.735)	(0.145)	(0.554)	(0.491)
LDL-C (mg/dL)	−0.040	−0.129	−0.417	−0.238	−0.218
	(0.877)	(0.6202)	(0.096)	(0.357)	(0.399)
TG (mg/dL)	0.088	0.396	0.177	0.137	0.580
	(0.733)	(0.116)	(0.497)	(0.599)	(0.015 *)
TC/HDL-C (mg/dL)	0.103	−0.018	−0.392	−0.290	0.061
	(0.694)	(0.945)	(0.119)	(0.259)	(0.815)
TG/HDL-C (mg/dL)	0.232	0.338	0.048	0.104	0.563
	(0.371)	(0.184)	(0.856)	(0.690)	(0.019 *)
LDL-C/HDL-C (mg/dL)	0.070	−0.147	−0.481	−0.355	−0.117
	(0.788)	(0.577)	(0.051)	(0.162)	(0.654)
TyG index (mg/dL)	0.162	0.142	0.059	0.212	0.412
	(0.536)	(0.586)	(0.823)	(0.415)	(0.100)
Albumin (g/dL)	0.061	0.276	−0.235	0.171	0.272
	(0.815)	(0.283)	(0.364)	(0.513)	(0.292)
Amylase, pancreatic (U/L)	0.218	−0.083	−0.193	−0.434	−0.085
	(0.401	(0.753)	(0.458)	(0.082)	(0.747)
Protein, total (g/dL)	0.103	0.578	−0.055	0.241	0.514
	(0.696)	(0.015 *)	(0.835)	(0.352)	(0.035 *)
Uric acid (mg/dL)	0.500	0.335	−0.327	−0.060	0.406
	(0.041 *)	(0.189)	(0.199)	(0.820)	(0.106)
Urea (mg/dL)	−0.405	−0.072	−0.188	−0.326	−0.078
	(0.107)	(0.785)	(0.469)	(0.202)	(0.764)
Creatinine (mg/dL)	0.143	0.056	−0.513	−0.396	0.100
	(0.585)	(0.831)	(0.035 *)	(0.116)	(0.702)

Correlation coefficient and *p*-value given in brackets below are shown. * indicates significant correlations (with *p*-values in bold; *p* < 0.05). FM, fat mass; SMM, skeletal muscle mass; VFA, visceral fat area; IL-6, interleukin-6; CRP, C-reactive protein; TC, total cholesterol; HDL-C, high-density lipoprotein cholesterol, LDL-C, low-density lipoprotein cholesterol; TG, triglyceride; TC/HDL-C, total cholesterol/high-density lipoprotein cholesterol ratio; TG/HDL-C, triglyceride/high-density lipoprotein cholesterol ratio; LDL-C/HDL-C, low-density lipoprotein cholesterol/high-density lipoprotein cholesterol ratio; TyG index, triglyceride-glucose index.

## Data Availability

The raw data supporting the conclusions of this article will be made available by the authors on reasonable request.

## References

[1] NCD Risk Factor Collaboration (NCD-RisC) (2017). Worldwide Trends in Body-Mass Index, Underweight, Overweight, and Obesity from 1975 to 2016: A Pooled Analysis of 2416 Population-Based Measurement Studies in 128.9 Million Children, Adolescents, and Adults. Lancet.

[2] Guthold R., Stevens G.A., Riley L.M., Bull F.C. (2018). Worldwide Trends in Insufficient Physical Activity from 2001 to 2016: A Pooled Analysis of 358 Population-Based Surveys with 1·9 Million Participants. Lancet Glob. Health.

[3] Gleeson M., Bishop N.C., Stensel D.J., Lindley M.R., Mastana S.S., Nimmo M.A. (2011). The Anti-Inflammatory Effects of Exercise: Mechanisms and Implications for the Prevention and Treatment of Disease. Nat. Rev. Immunol..

[4] Phelps N.H., Singleton R.K., Zhou B., Heap R.A., Mishra A., Bennett J.E., Paciorek C.J., Lhoste V.P., Carrillo-Larco R.M., Stevens G.A. (2024). Worldwide Trends in Underweight and Obesity from 1990 to 2022: A Pooled Analysis of 3663 Population-Representative Studies with 222 Million Children, Adolescents, and Adults. Lancet.

[5] Lobstein T., Jackson-Leach R., Powis J., Brinsden H., Gray M. (2023). World Obesity Atlas 2023.

[6] Mathis D., Shoelson S.E. (2011). Immunometabolism: An Emerging Frontier. Nat. Rev. Immunol..

[7] Doyle S.L., Donohoe C.L., Lysaght J., Reynolds J.V. (2012). Visceral Obesity, Metabolic Syndrome, Insulin Resistance and Cancer. Proc. Nutr. Soc..

[8] Silveira E.A., Kliemann N., Noll M., Sarrafzadegan N., de Oliveira C. (2021). Visceral Obesity and Incident Cancer and Cardiovascular Disease: An Integrative Review of the Epidemiological Evidence. Obes. Rev..

[9] Riaz H., Khan M.S., Siddiqi T.J., Usman M.S., Shah N., Goyal A., Khan S.S., Mookadam F., Krasuski R.A., Ahmed H. (2018). Association Between Obesity and Cardiovascular Outcomes. JAMA Netw. Open.

[10] Aparecida Silveira E., Vaseghi G., de Carvalho Santos A.S., Kliemann N., Masoudkabir F., Noll M., Mohammadifard N., Sarrafzadegan N., de Oliveira C. (2020). Visceral Obesity and Its Shared Role in Cancer and Cardiovascular Disease: A Scoping Review of the Pathophysiology and Pharmacological Treatments. Int. J. Mol. Sci..

[11] Gkavogiannakis N.A., Tsoporis J.N., Drosatos I.-A., Tsirebolos G., Izhar S., Sakadakis E., Triantafyllis A.S., Parker T.G., Kalogiros L.A., Leong-Poi H. (2023). Emergent Inflammatory Markers and Echocardiographic Indices in Patients with Bronchial Asthma. Biomolecules.

[12] Greenberg A.S., McDaniel M.L. (2002). Identifying the Links between Obesity, Insulin Resistance and Β-cell Function: Potential Role of Adipocyte-derived Cytokines in the Pathogenesis of Type 2 Diabetes. Eur. J. Clin. Investig..

[13] Henson J., Yates T., Edwardson C.L., Khunti K., Talbot D., Gray L.J., Leigh T.M., Carter P., Davies M.J. (2013). Sedentary Time and Markers of Chronic Low-Grade Inflammation in a High Risk Population. PLoS ONE.

[14] Wilmot E.G., Edwardson C.L., Achana F.A., Davies M.J., Gorely T., Gray L.J., Khunti K., Yates T., Biddle S.J.H. (2012). Sedentary Time in Adults and the Association with Diabetes, Cardiovascular Disease and Death: Systematic Review and Meta-Analysis. Diabetologia.

[15] Rodas L., Riera-Sampol A., Aguilo A., Martínez S., Tauler P. (2020). Effects of Habitual Caffeine Intake, Physical Activity Levels, and Sedentary Behavior on the Inflammatory Status in a Healthy Population. Nutrients.

[16] Saunders T.J., Larouche R., Colley R.C., Tremblay M.S. (2012). Acute Sedentary Behaviour and Markers of Cardiometabolic Risk: A Systematic Review of Intervention Studies. J. Nutr. Metab..

[17] Tremblay M.S., Aubert S., Barnes J.D., Saunders T.J., Carson V., Latimer-Cheung A.E., Chastin S.F.M., Altenburg T.M., Chinapaw M.J.M., Participants S.T.C.P. (2017). Sedentary Behavior Research Network (SBRN)—Terminology Consensus Project Process and Outcome. Int. J. Behav. Nutr. Phys. Act..

[18] Patterson R., McNamara E., Tainio M., de Sá T.H., Smith A.D., Sharp S.J., Edwards P., Woodcock J., Brage S., Wijndaele K. (2018). Sedentary Behaviour and Risk of All-Cause, Cardiovascular and Cancer Mortality, and Incident Type 2 Diabetes: A Systematic Review and Dose Response Meta-Analysis. Eur. J. Epidemiol..

[19] Ekelund U., Steene-Johannessen J., Brown W.J., Fagerland M.W., Owen N., Powell K.E., Bauman A., Lee I.M., Lancet Physical Activity Series 2 Executive C., Lancet Sedentary Behaviour Working G. (2016). Does Physical Activity Attenuate, or Even Eliminate, the Detrimental Association of Sitting Time with Mortality? A Harmonised Meta-Analysis of Data from More than 1 Million Men and Women. Lancet.

[20] Dunstan D.W., Dogra S., Carter S.E., Owen N. (2021). Sit Less and Move More for Cardiovascular Health: Emerging Insights and Opportunities. Nat. Rev. Cardiol..

[21] Bennie J.A., Chau J.Y., van der Ploeg H.P., Stamatakis E., Do A., Bauman A. (2013). The Prevalence and Correlates of Sitting in European Adults—A Comparison of 32 Eurobarometer-Participating Countries. Int. J. Behav. Nutr. Phys. Act..

[22] Edelmann D., Pfirrmann D., Heller S., Dietz P., Reichel J.L., Werner A.M., Schäfer M., Tibubos A.N., Deci N., Letzel S. (2022). Physical Activity and Sedentary Behavior in University Students-The Role of Gender, Age, Field of Study, Targeted Degree, and Study Semester. Front. Public Health.

[23] Castro O., Bennie J., Vergeer I., Bosselut G., Biddle S.J.H. (2020). How Sedentary Are University Students? A Systematic Review and Meta-Analysis. Prev. Sci..

[24] Hoffmann S.W., Schierbauer J., Zimmermann P., Voit T., Grothoff A., Wachsmuth N., Rössler A., Lackner H.K., Moser O. (2024). Effects of Light-intensity Physical Activity on Cardiometabolic Parameters in Young Adults with Overweight and Obesity: The SED-ACT Randomized Controlled Crossover Trial. Diabetes Obes. Metab..

[25] Pedersen B.K., Febbraio M.A. (2008). Muscle as an Endocrine Organ: Focus on Muscle-Derived Interleukin-6. Physiol. Rev..

[26] Pedersen B.K., Fischer C.P. (2007). Beneficial Health Effects of Exercise—The Role of IL-6 as a Myokine. Trends Pharmacol. Sci..

[27] Mujumdar P.P., Duerksen-Hughes P.J., Firek A.F., Hessinger D.A. (2011). Long-Term, Progressive, Aerobic Training Increases Adiponectin in Middle-Aged, Overweight, Untrained Males and Females. Scand. J. Clin. Lab. Investig..

[28] Zunner B.E.M., Wachsmuth N.B., Eckstein M.L., Scherl L., Schierbauer J.R., Haupt S., Stumpf C., Reusch L., Moser O. (2022). Myokines and Resistance Training: A Narrative Review. Int. J. Mol. Sci..

[29] Plomgaard P., Bouzakri K., Krogh-Madsen R., Mittendorfer B., Zierath J.R., Pedersen B.K. (2005). Tumor Necrosis Factor-α Induces Skeletal Muscle Insulin Resistance in Healthy Human Subjects via Inhibition of Akt Substrate 160 Phosphorylation. Diabetes.

[30] Balder J.W., de Vries J.K., Nolte I.M., Lansberg P.J., Kuivenhoven J.A., Kamphuisen P.W. (2017). Lipid and Lipoprotein Reference Values from 133,450 Dutch Lifelines Participants: Age- and Gender-Specific Baseline Lipid Values and Percentiles. J. Clin. Lipidol..

[31] Kannel W.B., Vasan R.S., Keyes M.J., Sullivan L.M., Robins S.J. (2008). Usefulness of the Triglyceride–High-Density Lipoprotein Versus the Cholesterol–High-Density Lipoprotein Ratio for Predicting Insulin Resistance and Cardiometabolic Risk (from the Framingham Offspring Cohort). Am. J. Cardiol..

[32] Stadler J.T., Marsche G. (2020). Obesity-Related Changes in High-Density Lipoprotein Metabolism and Function. Int. J. Mol. Sci..

[33] Nussbaumerova B., Rosolova H. (2023). Obesity and Dyslipidemia. Curr. Atheroscler. Rep..

[34] van Mil D., Kieneker L.M., Evers-Roeten B., Thelen M.H.M., de Vries H., Hemmelder M.H., Dorgelo A., van Etten R.W., Heerspink H.J.L., Gansevoort R.T. (2023). Participation Rate and Yield of Two Home-Based Screening Methods to Detect Increased Albuminuria in the General Population in the Netherlands (THOMAS): A Prospective, Randomised, Open-Label Implementation Study. Lancet.

[35] Dai Y., Quan J., Xiong L., Luo Y., Yi B. (2022). Probiotics Improve Renal Function, Glucose, Lipids, Inflammation and Oxidative Stress in Diabetic Kidney Disease: A Systematic Review and Meta-Analysis. Ren. Fail..

[36] Harriss D.J., MacSween A., Atkinson G. (2019). Ethical Standards in Sport and Exercise Science Research: 2020 Update. Int. J. Sports Med..

[37] Urbaniak G., Plous S. (2013). Research Randomizer (Version 4.0). https://www.randomizer.org/.

[38] Dwan K., Li T., Altman D.G., Elbourne D. (2019). CONSORT 2010 Statement: Extension to Randomised Crossover Trials. BMJ.

[39] Schleicher E., Gerdes C., Petersmann A., Müller-Wieland D., Müller U.A., Freckmann G., Heinemann L., Nauck M., Landgraf R. (2022). Definition, Classification and Diagnosis of Diabetes Mellitus. Exp. Clin. Endocrinol. Diabetes.

[40] Crespo N.C., Mullane S.L., Zeigler Z.S., Buman M.P., Gaesser G.A. (2016). Effects of Standing and Light-Intensity Walking and Cycling on 24-h Glucose. Med. Sci. Sports Exerc..

[41] Zeigler Z.S., Mullane S.L., Crespo N.C., Buman M.P., Gaesser G.A. (2016). Effects of Standing and Light-Intensity Activity on Ambulatory Blood Pressure. Med. Sci. Sports Exerc..

[42] Thorp A.A., Kingwell B.A., Owen N., Dunstan D.W. (2014). Breaking up Workplace Sitting Time with Intermittent Standing Bouts Improves Fatigue and Musculoskeletal Discomfort in Overweight/Obese Office Workers. Occup. Environ. Med..

[43] Manning P.J., Sutherland W.H.F., McGrath M.M., De Jong S.A., Walker R.J., Williams M.J.A. (2008). Postprandial Cytokine Concentrations and Meal Composition in Obese and Lean Women. Obesity.

[44] Tao L.-C., Xu J., Wang T., Hua F., Li J.-J. (2022). Triglyceride-Glucose Index as a Marker in Cardiovascular Diseases: Landscape and Limitations. Cardiovasc. Diabetol..

[45] Araújo S.P., Juvanhol L.L., Bressan J., Hermsdorff H.H.M. (2022). Triglyceride Glucose Index: A New Biomarker in Predicting Cardiovascular Risk. Prev. Med. Rep..

[46] Derstine B.A., Holcombe S.A., Ross B.E., Wang N.C., Wang S.C., Su G.L. (2022). Healthy US Population Reference Values for CT Visceral Fat Measurements and the Impact of IV Contrast, HU Range, and Spinal Levels. Sci. Rep..

[47] Achamrah N., Colange G., Delay J., Rimbert A., Folope V., Petit A., Grigioni S., Déchelotte P., Coëffier M. (2018). Comparison of Body Composition Assessment by DXA and BIA According to the Body Mass Index: A Retrospective Study on 3655 Measures. PLoS ONE.

[48] Ference B.A., Ginsberg H.N., Graham I., Ray K.K., Packard C.J., Bruckert E., Hegele R.A., Krauss R.M., Raal F.J., Schunkert H. (2017). Low-Density Lipoproteins Cause Atherosclerotic Cardiovascular Disease. 1. Evidence from Genetic, Epidemiologic, and Clinical Studies. A Consensus Statement from the European Atherosclerosis Society Consensus Panel. Eur. Heart J..

[49] Grundy S.M., Stone N.J., Bailey A.L., Beam C., Birtcher K.K., Blumenthal R.S., Braun L.T., de Ferranti S., Faiella-Tommasino J., Forman D.E. (2019). 2018 AHA/ACC/AACVPR/AAPA/ABC/ACPM/ADA/AGS/APhA/ASPC/NLA/PCNA Guideline on the Management of Blood Cholesterol: Executive Summary. J. Am. Coll. Cardiol..

[50] Fischer C.P. (2006). Interleukin-6 in Acute Exercise and Training: What Is the Biological Relevance?. Exerc. Immunol. Rev.

[51] Ulven S.M., Foss S.S., Skjølsvik A.M., Stadheim H.K., Myhrstad M.C.W., Raael E., Sandvik M., Narverud I., Andersen L.F., Jensen J. (2015). An Acute Bout of Exercise Modulate the Inflammatory Response in Peripheral Blood Mononuclear Cells in Healthy Young Men. Arch. Physiol. Biochem..

[52] Son W.-H., Park H.-T., Jeon B.H., Ha M.-S. (2023). Moderate Intensity Walking Exercises Reduce the Body Mass Index and Vascular Inflammatory Factors in Postmenopausal Women with Obesity: A Randomized Controlled Trial. Sci. Rep..

[53] Moore K.W., de Waal Malefyt R., Coffman R.L., O’Garra A. (2001). Interleukin-10 and the Interleukin-10 Receptor. Annu. Rev. Immunol..

[54] Park H.S., Park J.Y., Yu R. (2005). Relationship of Obesity and Visceral Adiposity with Serum Concentrations of CRP, TNF-α and IL-6. Diabetes Res. Clin. Pract..

[55] Panagiotakos D., Pitsavos C., Yannakoulia M., Chrysohoou C., Stefanadis C. (2005). The Implication of Obesity and Central Fat on Markers of Chronic Inflammation: The ATTICA Study. Atherosclerosis.

[56] Lombardo M., Feraco A., Bellia C., Prisco L., D’Ippolito I., Padua E., Storz M.A., Lauro D., Caprio M., Bellia A. (2022). Influence of Nutritional Status and Physical Exercise on Immune Response in Metabolic Syndrome. Nutrients.

[57] Maylor B.D., Zakrzewski-Fruer J.K., Orton C.J., Bailey D.P. (2018). Beneficial Postprandial Lipaemic Effects of Interrupting Sedentary Time with High-Intensity Physical Activity versus a Continuous Moderate-Intensity Physical Activity Bout: A Randomised Crossover Trial. J. Sci. Med. Sport.

[58] Duvivier B.M.F.M., Schaper N.C., Bremers M.A., van Crombrugge G., Menheere P.P.C.A., Kars M., Savelberg H.H.C.M. (2013). Minimal Intensity Physical Activity (Standing and Walking) of Longer Duration Improves Insulin Action and Plasma Lipids More than Shorter Periods of Moderate to Vigorous Exercise (Cycling) in Sedentary Subjects When Energy Expenditure Is Comparable. PLoS ONE.

[59] Nieste I., Franssen W.M.A., Duvivier B.M.F.M., Spaas J., Savelberg H.H.C.M., Eijnde B.O. (2023). Replacing Sitting with Light-Intensity Physical Activity throughout the Day versus 1 Bout of Vigorous-Intensity Exercise: Similar Cardiometabolic Health Effects in Multiple Sclerosis. A Randomised Cross-over Study. Disabil. Rehabil..

[60] Prasertsri P., Phoemsapthawee J., Kuamsub S., Poolpol K., Boonla O. (2022). Effects of Long-Term Regular Continuous and Intermittent Walking on Oxidative Stress, Metabolic Profile, Heart Rate Variability, and Blood Pressure in Older Adults with Hypertension. J. Environ. Public Health.

[61] Chiang T.-L., Chen C., Hsu C.-H., Lin Y.-C., Wu H.-J. (2019). Is the Goal of 12,000 Steps per Day Sufficient for Improving Body Composition and Metabolic Syndrome? The Necessity of Combining Exercise Intensity: A Randomized Controlled Trial. BMC Public Health.

[62] Champion R.B., Smith L.R., Smith J., Hirlav B., Maylor B.D., White S.L., Bailey D.P. (2018). Reducing Prolonged Sedentary Time Using a Treadmill Desk Acutely Improves Cardiometabolic Risk Markers in Male and Female Adults. J. Sports Sci..

[63] Snyder C.K., Lapidus J.A., Cawthon P.M., Dam T.L., Sakai L.Y., Marshall L.M. (2012). Serum Albumin in Relation to Change in Muscle Mass, Muscle Strength, and Muscle Power in Older Men. J. Am. Geriatr. Soc..

[64] Reijnierse E.M., Trappenburg M.C., Leter M.J., Sipilä S., Stenroth L., Narici M.V., Hogrel J.Y., Butler-Browne G., McPhee J.S., Pääsuke M. (2015). Serum Albumin and Muscle Measures in a Cohort of Healthy Young and Old Participants. AGE.

[65] Visser M., Kritchevsky S.B., Newman A.B., Goodpaster B.H., Tylavsky F.A., Nevitt M.C., Harris T.B. (2005). Lower Serum Albumin Concentration and Change in Muscle Mass: The Health, Aging and Body Composition Study. Am. J. Clin. Nutr..

[66] Thompson L.V. (2009). Age-Related Muscle Dysfunction. Exp. Gerontol..

[67] Lai K.-M.V., Gonzalez M., Poueymirou W.T., Kline W.O., Na E., Zlotchenko E., Stitt T.N., Economides A.N., Yancopoulos G.D., Glass D.J. (2004). Conditional Activation of Akt in Adult Skeletal Muscle Induces Rapid Hypertrophy. Mol. Cell. Biol..

[68] Hou Y., Ma R., Gao S., Kaudimba K.K., Yan H., Liu T., Wang R. (2021). The Effect of Low and Moderate Exercise on Hyperuricemia: Protocol for a Randomized Controlled Study. Front. Endocrinol..

[69] Liu R., Han C., Wu D., Xia X., Gu J., Guan H., Shan Z., Teng W. (2015). Prevalence of Hyperuricemia and Gout in Mainland China from 2000 to 2014: A Systematic Review and Meta-Analysis. BioMed Res. Int..

[70] Park D.Y., Kim Y.S., Ryu S.H., Jin Y.S. (2019). The Association between Sedentary Behavior, Physical Activity and Hyperuricemia. Vasc. Health Risk Manag..

[71] Chen J.-H., Wen C.P., Wu S.B., Lan J.-L., Tsai M.K., Tai Y.-P., Lee J.H., Hsu C.C., Tsao C.K., Wai J.P.M. (2015). Attenuating the Mortality Risk of High Serum Uric Acid: The Role of Physical Activity Underused. Ann. Rheum. Dis..

